# Author Correction: Strong synergy between gold nanoparticles and cobalt porphyrin induces highly efficient photocatalytic hydrogen evolution

**DOI:** 10.1038/s41467-024-52361-y

**Published:** 2024-09-10

**Authors:** Huixiang Sheng, Jin Wang, Juhui Huang, Zhuoyao Li, Guozhang Ren, Linrong Zhang, Liuyingzi Yu, Mengshuai Zhao, Xuehui Li, Gongqiang Li, Ning Wang, Chen Shen, Gang Lu

**Affiliations:** 1 Key Laboratory of Flexible Electronics (KLoFE) and Institute of Advanced Materials (IAM), Nanjing Tech University (NanjingTech), 30 South Puzhu Road, Nanjing, 211816 China; 2 School of Physics, University of Electronic Science and Technology of China, Chengdu, 610054 P. R. China; 3 Institute of Materials Science, Technical University of Darmstadt, Darmstadt, 64287 Germany; 4 National Laboratory of Solid State Microstructures, Nanjing University, Nanjing, 210093 China

Correction to: *Nature Communications* 10.1038/s41467-023-37271-9, published online 18 March 2023

In this article, fig. 1d and fig.1e X axis read ‘Binging Energy’ and should have read ‘Binding Energy’. The original article has been corrected.

