# Supplementary material for: Strong synergy between gold nanoparticles and cobalt porphyrin induces highly efficient photocatalytic hydrogen evolution
Source: Nat Commun. 2023 Mar 18;14:1528. doi: 10.1038/s41467-023-37271-9 (PMC10024688; doi:10.1038/s41467-023-37271-9)
Supplement: Supplementary file 1 — Supplementary Information [file 41467_2023_37271_MOESM1_ESM.pdf]

Supplementary Information

Strong synergy between gold nanoparticles and cobalt porphyrin induces highly efficient photocatalytic

Huixiang Sheng,[1] Jin Wang,[1] Juhui Huang,[1] Zhuoyao Li,[1] Guozhang Ren,[1] Linrong Zhang,[1] Liuyingzi Yu,[1] Mengshuai Zhao,[1] Xuehui Li,[1] Gongqiang Li,[1] Ning Wang,[2] Chen Shen,[3] Gang Lu*[1,4]

[1]Key Laboratory of Flexible Electronics (KLoFE) and Institute of Advanced Materials (IAM), Nanjing Tech University (NanjingTech), 30 South Puzhu Road, Nanjing 211816, China

[2]School of Physics, University of Electronic Science and Technology of China, Chengdu 610054, P. R. China

[3]Institute of Materials Science, Technical University of Darmstadt, Darmstadt 64287, Germany

[4]National Laboratory of Solid State Microstructures, Nanjing University, Nanjing 210093, China

* Corresponding author: Gang Lu, iamglv@njtech.edu.cn

Table of Contents

Supplementary Fig. 1 Schematic diagram showing the linking of AuNPs with CoTPyP molecules. S4

Supplementary Fig. 2 UV−Vis extinction spectrum of the CoTPyP (2 μM) and AuNP@CoTPyP (CoTPyP concentration = 2 μM). S4

Supplementary Fig. 3 Raman spectra (bottom curve) and SERS (top curve) spectra of CoTPyP. S5

Supplementary Fig. 4 TEM images of AuNP@CoTPyP (a) initially and (b) after 45 hours of reaction. S5

Supplementary Fig. 5 UV–Vis spectrum of AuNP@CoTPyP initially and after 45 hours of reaction. S6

Supplementary Fig. 6 XPS spectra of (a) Au 4f, (b) N 1s initially and after 45 hours of reaction. S6

Supplementary Fig. 7 UV−Vis extinction spectrum and the photograph of the AuNP@CoTPyP prepared by using 2 nM CoTPyP solution and TEM images of AuNP@CoTPyP (CoTPyP concentration = 2 nM). S6

Supplementary Fig. 8 TEM images of AuNP@CoTPyP (CoTPyP concentration = 20 nM). S7

Supplementary Fig. 9 HER production and TON of the washed AuNP@CoTPyP samples prepared at different concentrations of CoTPyP. S7

Supplementary Fig. 10 SEM image and UV−Vis extinction spectrum of AuNRs.S7

Supplementary Fig. 11 UV−Vis extinction spectrum of AuNRs and AuNR@CoTPyP and Photocatalytic HER curve of AuNR@CoTPyP. S8

Supplementary Fig. 12 STEM image of AuNR@CoTPyP and corresponding EDS element mapping images. S8

Supplementary Fig. 13 SEM image and UV−Vis extinction spectrum of AgNP@CoTPyP and STEM image of AgNP@CoTPyP and corresponding EDS element mapping images. S9

Supplementary Fig. 14 Photocatalytic HER curve of AgNP@CoTPyP. S9

Supplementary Fig. 15 Single frame of the AuNP during SMFM and Reconstructed image of the catalytic active events (104 frames were acquired within 200 s). S10

Supplementary Fig. 16 UV−Vis extinction spectrum of AgNP@CoTPyP (CoTPyP concentration = 2 nM) and the HER rates under illuminations of monochromatic light. S10

Supplementary Fig. 17 FDTD simulations of the electromagnetic field enhancements. S11

Supplementary Fig. 18 Spectroelectrochemistry of CoTPyP. S11

Supplementary Fig. 19 Ultrafast transient absorption spectra of AuNP@CoTPyP at 800-1500 ps. S12

Supplementary Fig. 20 Photoluminescence spectra of CoTPyP (concentration is 2 μM) AuNP@CoTPyP (CoTPyP concentration is 2 μM). S12

Supplementary Fig. 21 Free energy diagram for the Volmer−Heyrovsky route and Volmer−Tafel pathway on AuNP@CoTPyP. S12

Supplementary Fig. 22 UV−Vis extinction spectrum of TPyP and CoTPyP. S13

Supplementary table 1 Photocatalytic HER rates of recently reported photocatalysts. S13

Supplementary table 2 ICP-OES analysis of the AuNP@CoTPyP structures prepared at different concentrations of CoTPyP. S14

Supplementary table 3 Frontier orbital energies for CoTPyP and AuNP@CoTPyP. S14

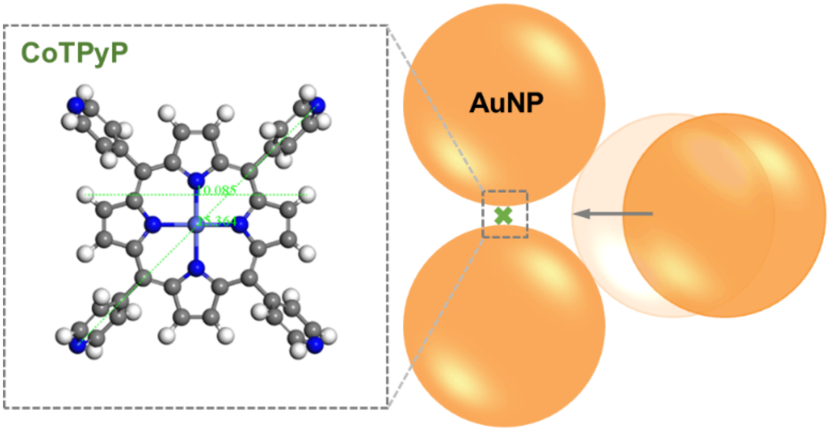

Supplementary Fig. 1. Schematic diagram showing the linking of AuNPs with CoTPyP molecules. The third AuNP (right one) cannot be linked to the CoTPyP molecule (marked green) between first and second AuNPs.

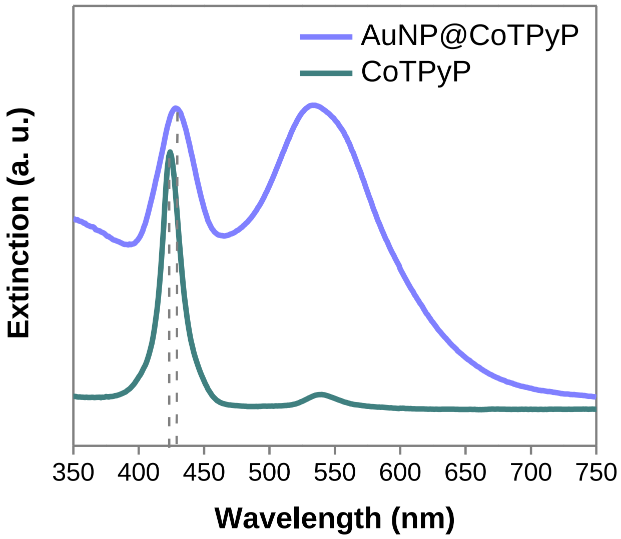

Supplementary Fig. 2. UV−Vis extinction spectrum of the CoTPyP (2 μM) and AuNP@CoTPyP (CoTPyP concentration = 2 μM).

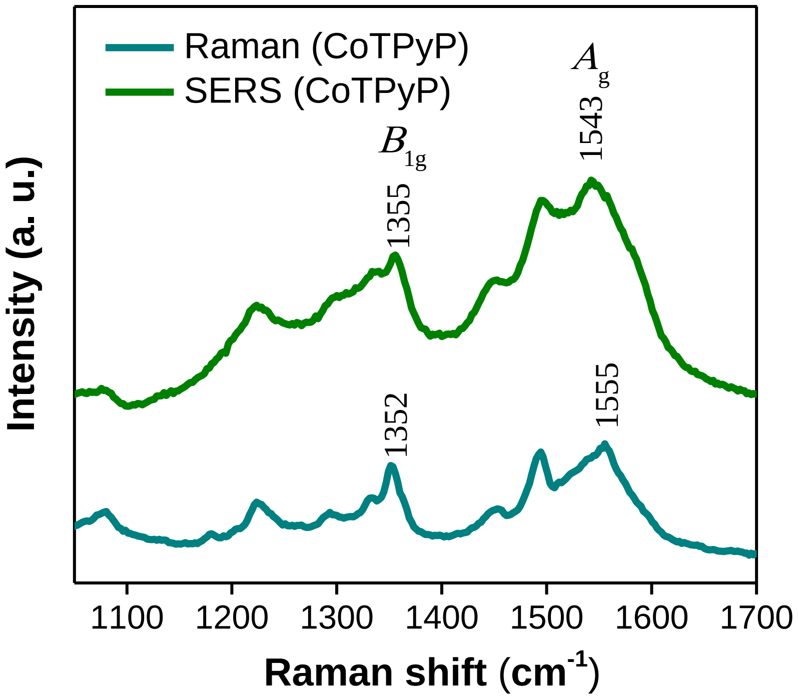

Supplementary Fig. 3. Raman spectra (bottom curve) and SERS (top curve) spectra of CoTPyP. The A_g and B_{1g} vibrational modes of CoTPyP were detected at 1555 and 1352 cm^{-1}, respectively. It is known that A_g mode arises from the $\upsilon(C_\beta C_\beta)$ stretching of the porphyrin ring together with a smaller contribution from the $\upsilon(C_\alpha C_m)$, while B_{1g} mode is a response of the pyrrole half-ring symmetric stretching modes. After being adsorbed onto AuNPs, B_{1g} vibrational mode of CoTPyP shifted to a higher wavenumber at 1355 cm^{-1}, A_g modes shifted to a lower wavenumber at 1543 cm^{-1}.

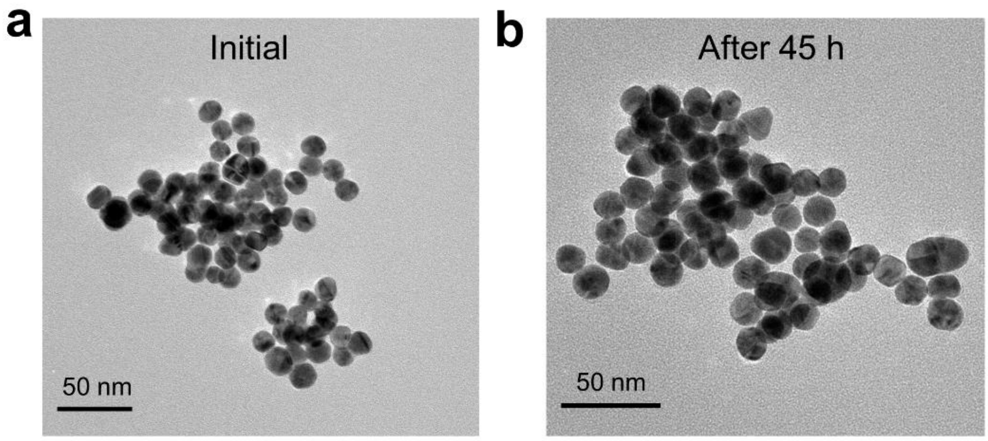

Supplementary Fig. 4. TEM images of AuNP@CoTPyP (a) initially and (b) after 45 hours of reaction.

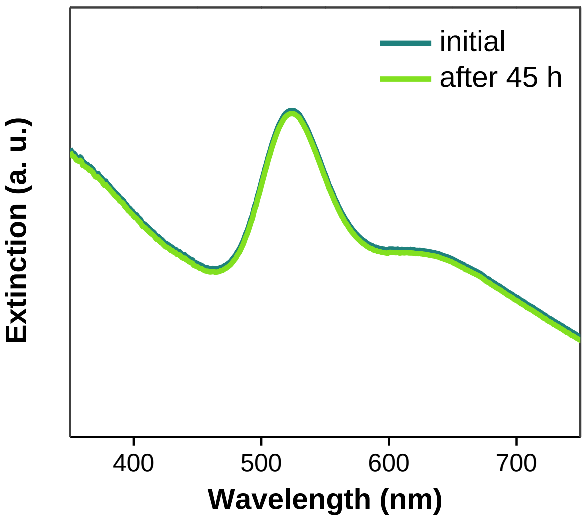

Supplementary Fig. 5. UV–Vis spectrum of AuNP@CoTPyP initially and after 45 hours of reaction.

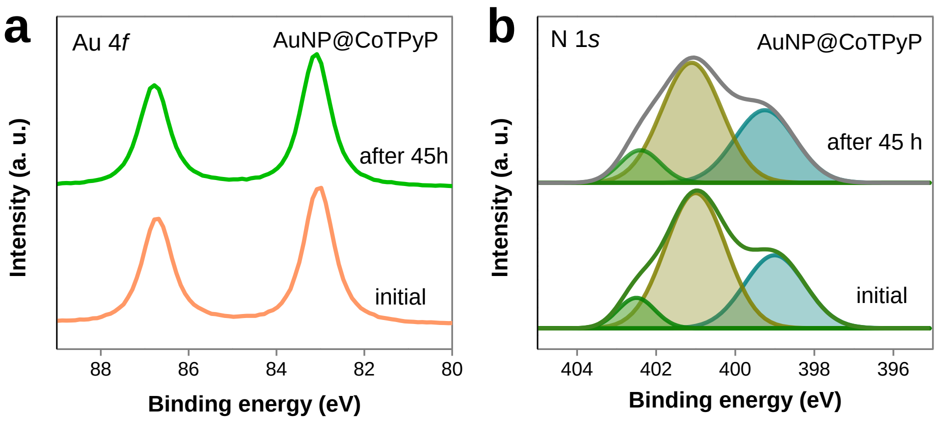

Supplementary Fig. 6 XPS spectra of (a) Au 4f, (b) N 1s initially and after 45 hours of reaction.

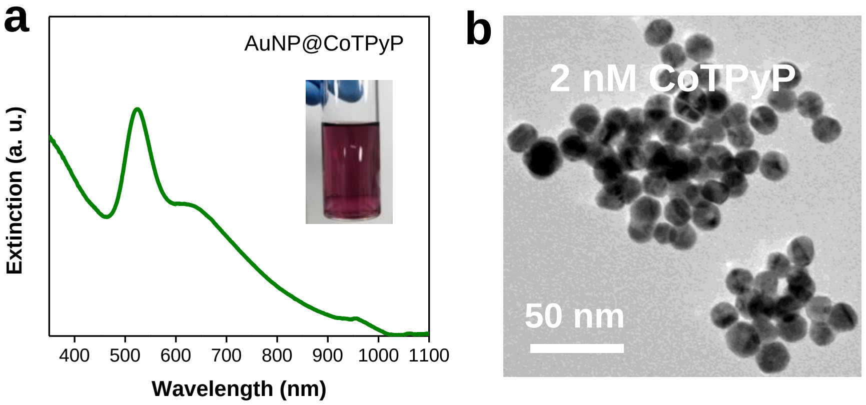

Supplementary Fig. 7. (a) UV−Vis extinction spectrum and the photograph of the AuNP@CoTPyP prepared by using 2 nM CoTPyP solution. (b) TEM images of AuNP@CoTPyP (CoTPyP concentration = 2 nM).

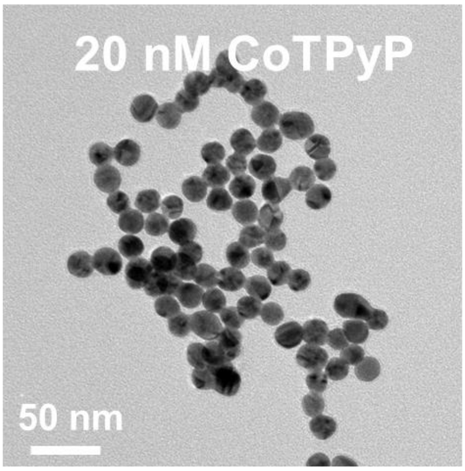

Supplementary Fig. 8. TEM images of AuNP@CoTPyP (CoTPyP concentration = 20 nM).

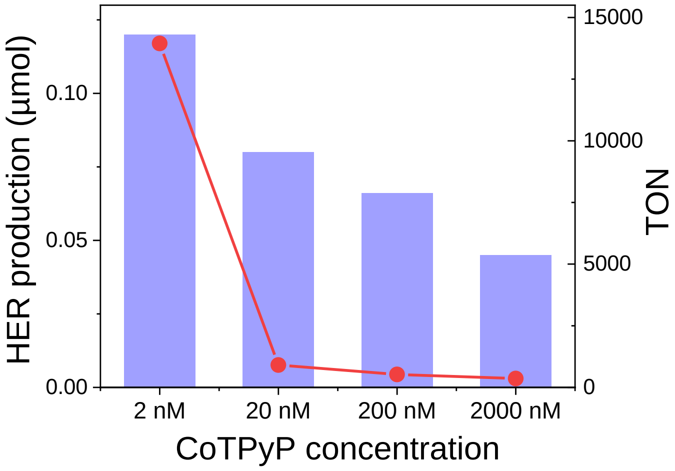

Supplementary Fig. 9. HER production and TON of the washed AuNP@CoTPyP samples prepared at different concentrations of CoTPyP.

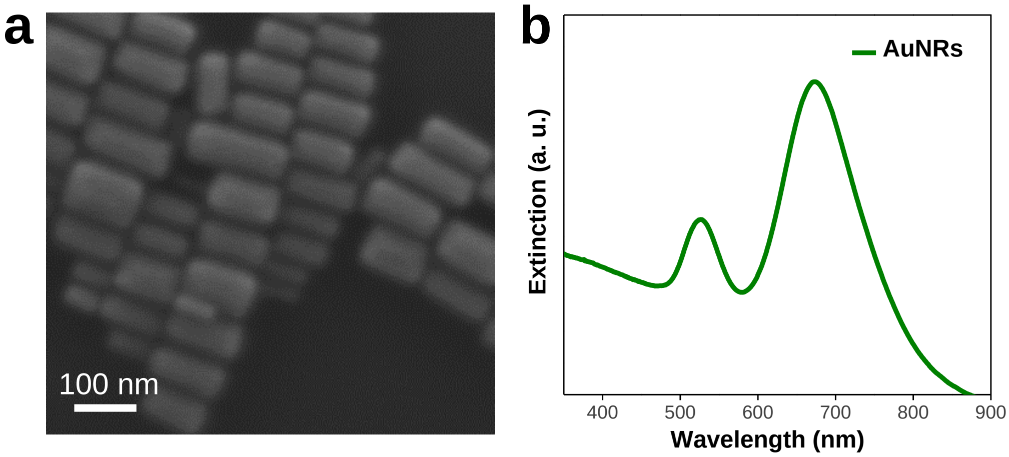

Supplementary Fig. 10. (a) SEM image of AuNRs. (b) UV−Vis extinction spectrum of AuNRs.

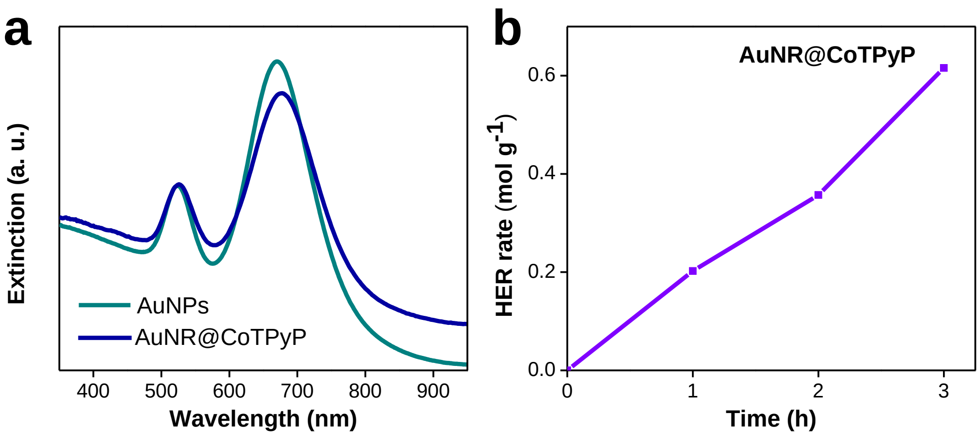

Supplementary Fig. 11. (a) UV−Vis extinction spectrum of AuNRs and AuNR@CoTPyP. (b) Photocatalytic HER curve of AuNR@CoTPyP.

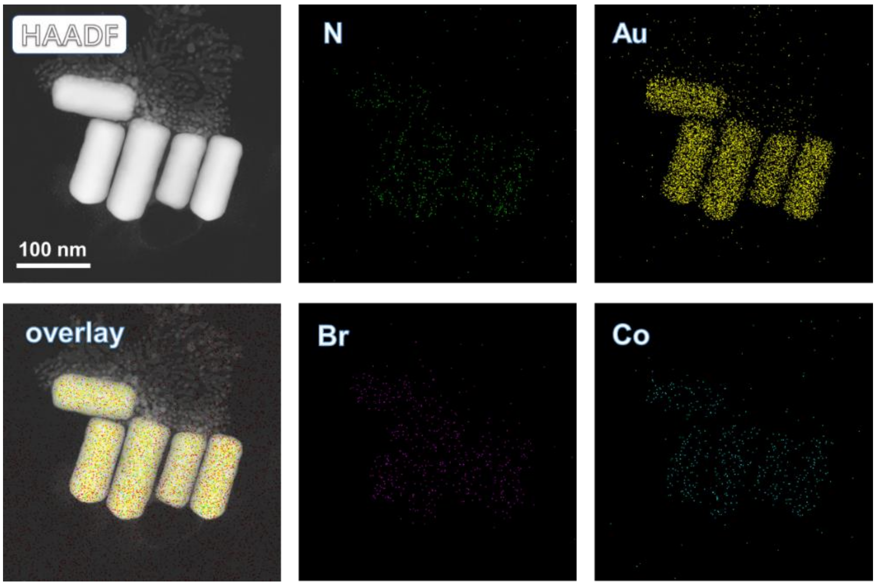

Supplementary Fig. 12. STEM image of AuNR@CoTPyP and corresponding EDS element mapping images.

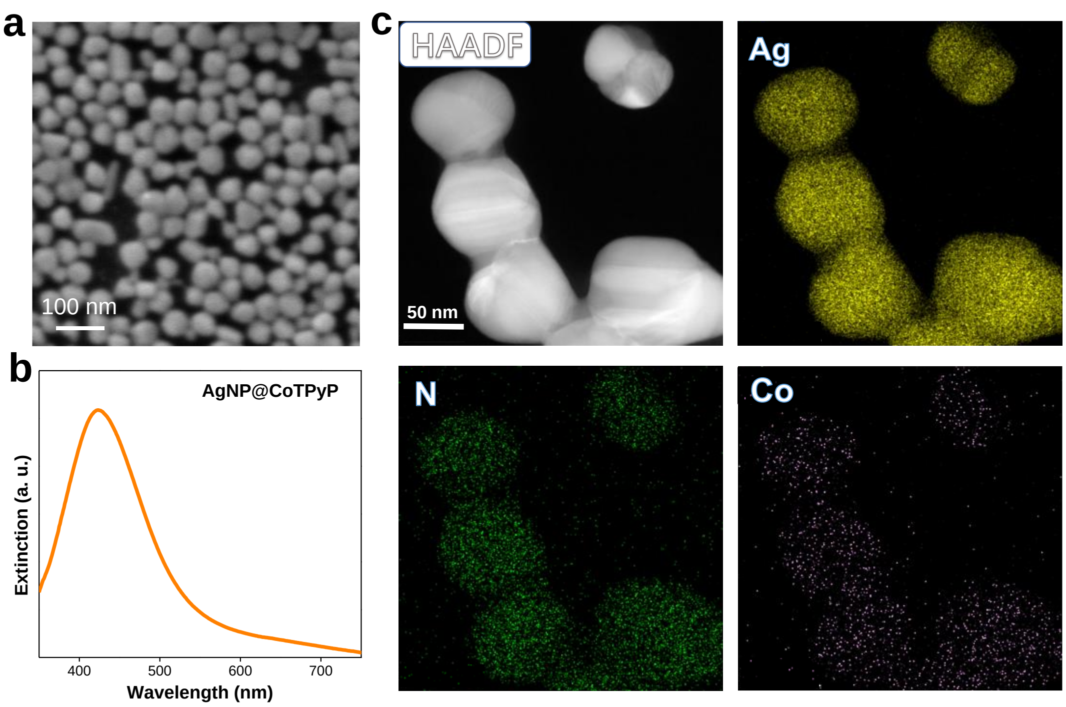

Supplementary Fig. 13. (a) SEM image of AgNP@CoTPyP. (b) UV−Vis extinction spectrum of AgNP@CoTPyP. (c) STEM image of AgNP@CoTPyP and corresponding EDS element mapping images.

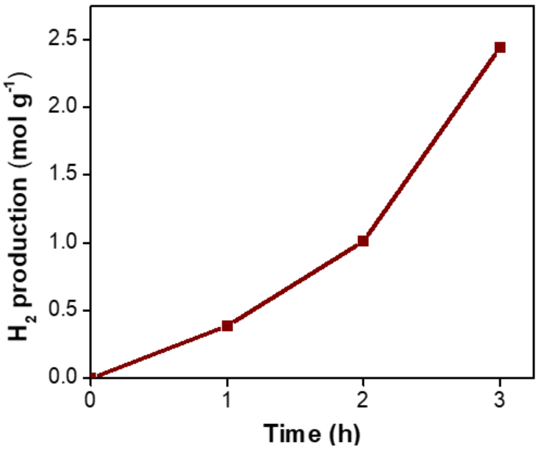

Supplementary Fig. 14. Photocatalytic HER curve of AgNP@CoTPyP.

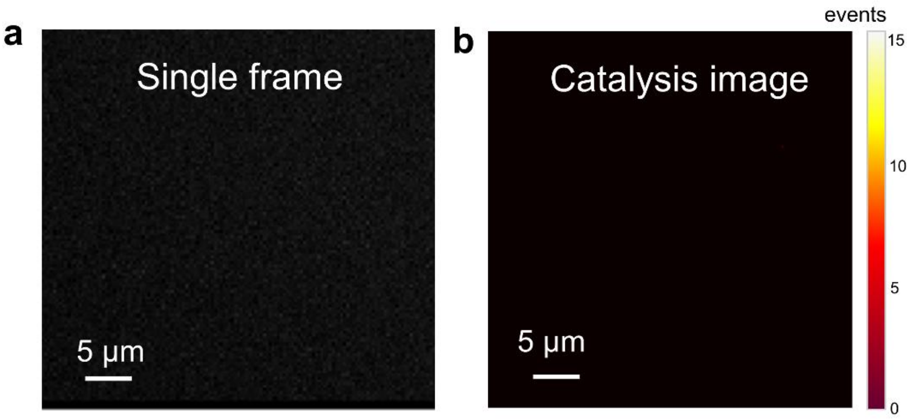

Supplementary Fig. 15. (a) Single frame of the AuNP during SMFM. (b) Reconstructed image of the catalytic active events (10^4 frames were acquired within 200 s).

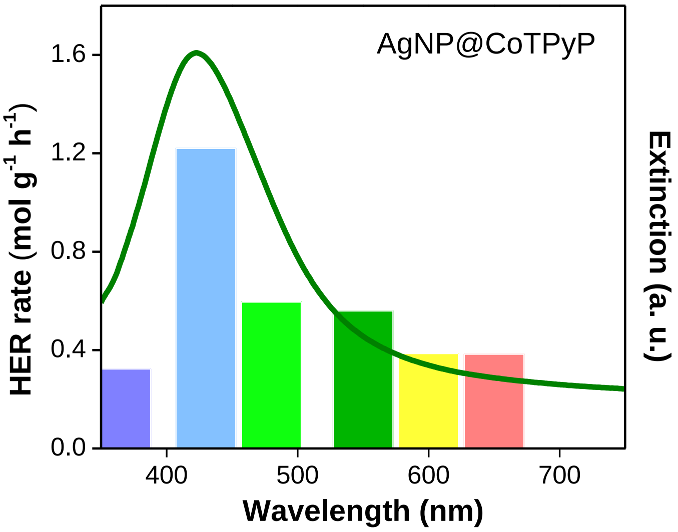

Supplementary Fig. 16. UV−Vis extinction spectrum of AgNP@CoTPyP (CoTPyP concentration = 2 nM) and the HER rates under illuminations of monochromatic light. The power was set as 5.2 W at all wavelengths.

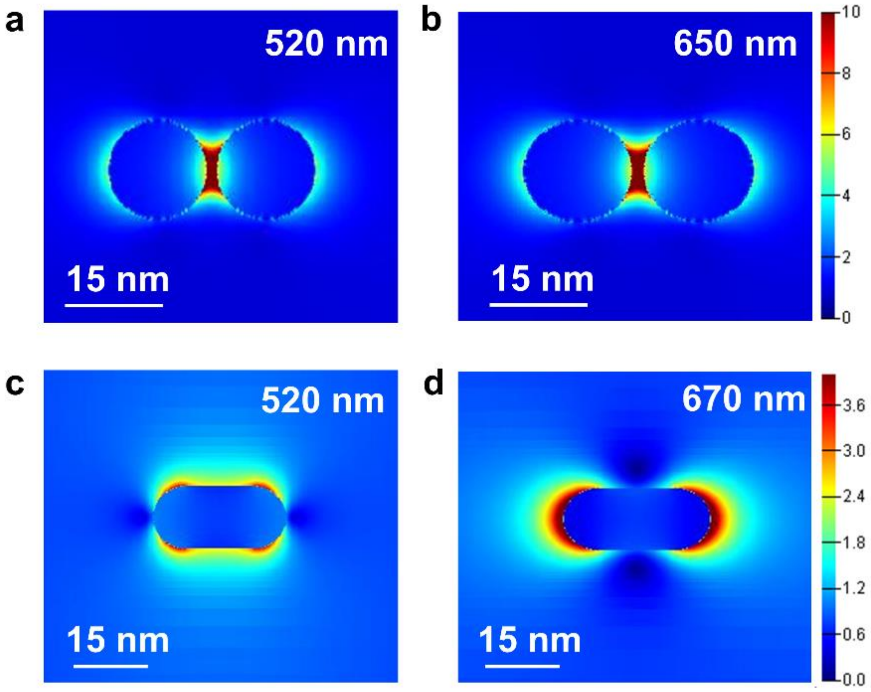

Supplementary Fig. 17. FDTD simulations of the electromagnetic field enhancements around (a) and (b) Au NP @CoTPyP aggregates under the excitation of 520 and 650 nm light, and (c) and (d) Au NR @CoTPyP under the excitation of 520 and 670nm light. (Diameter of Au NP = 15 nm; Length of Au NR = 50nm, Diameter of Au NR = 25 nm)

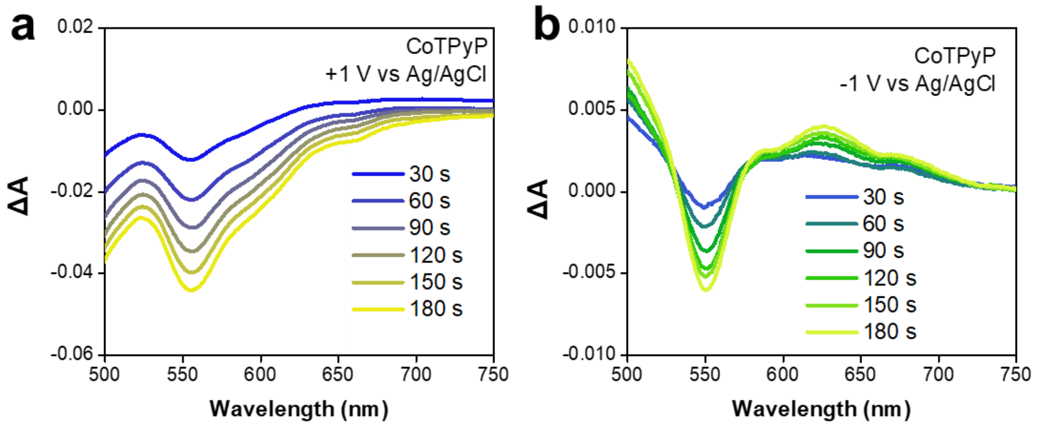

Supplementary Fig. 18. Spectroelectrochemistry of CoTPyP. UV-Vis differential absorption spectra of 2 μM CoTPyP in mixed solvent of CH_3CN and water (1:1) under (a) oxidation (1 V) and (b) reduction (-1 V) conditions. The solutions were bubbled with N_2 gas before the measurements and the potential was versus Ag/AgCl electrode.

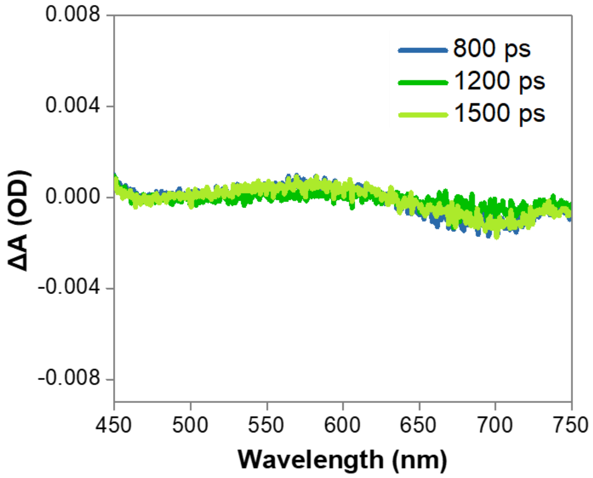

Supplementary Fig. 19. Ultrafast transient absorption spectra of AuNP@CoTPyP at 800-1500 ps.

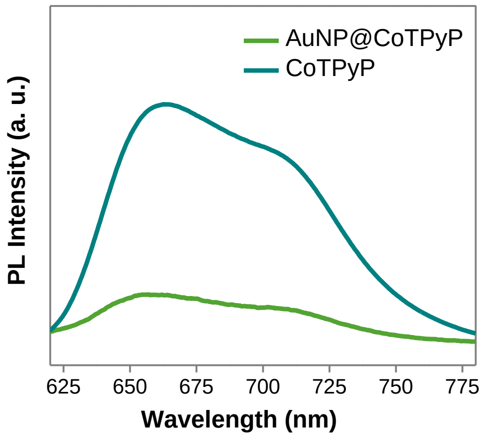

Supplementary Fig. 20. Photoluminescence spectra of CoTPyP (concentration is 2 μM) AuNP@CoTPyP (CoTPyP concentration is 2 μM).

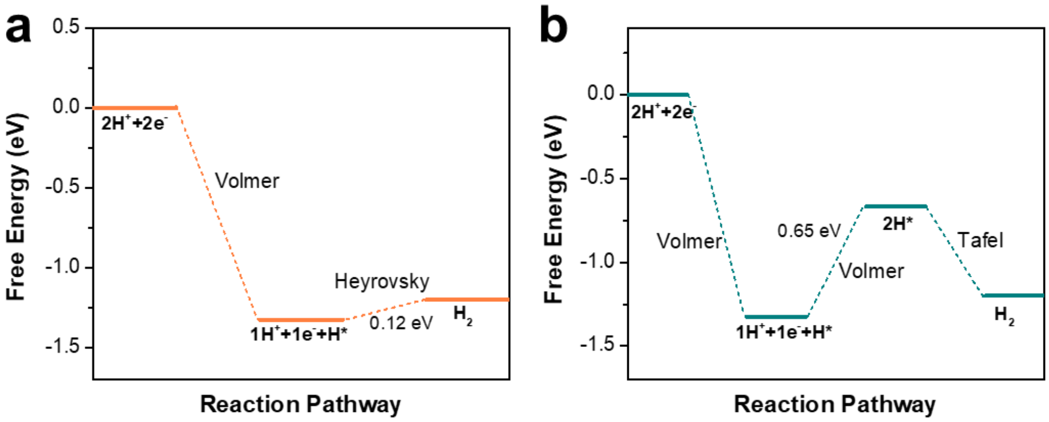

Supplementary Fig. 21. Free energy diagram for the (a) Volmer−Heyrovsky route and (b) Volmer−Tafel pathway on AuNP@CoTPyP.

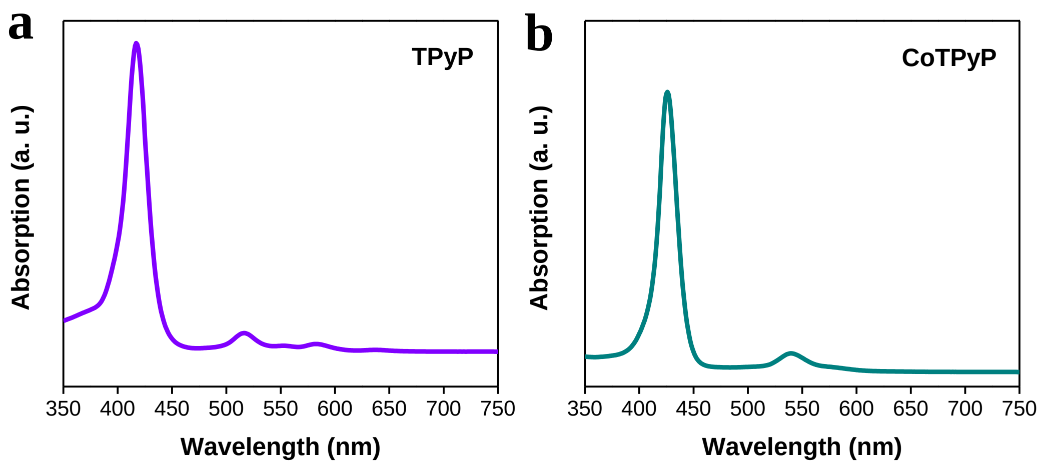

Supplementary Fig. 22. UV−Vis extinction spectrum of (a) TPyP and (b) CoTPyP.

Supplementary table 1 Photocatalytic HER rates of recently reported photocatalysts.

	**Production rate (mmol g^{-1}h^{-1})**	**Cyclic tests (h)**	**Light source (W)**	**Publication year**	**reference**
**AuNP@CoTPyP**	**3214**	**45**	**300**	**/**	**This work**
Au@UiOS@ZIS	39.1	30	300	2021	32
Ag SHIN-Au@CdS	191.2	/	0.35	2021	38
Ir-complex	17.8	12	175	2019	36
MTV-Ti-MOF/COF	13.98	120	300	2022	26
$CoWO_4/Mn_{0.47}Cd_{0.53}S$-25	41.53	16	300	2021	37
g-$C_3N_4/ZnIn_2S_4$	20.7	/	300	2022	27
$CdIn_2S_4$/CNFs/Co_4S_3	25.9	15	300	2021	29
CdS/NiO core-shell	243.9	18	225	2021	33
ZnDC(p-NI)PP/CoPyCl	35.7	50	1.5 sun	2021	25
Pt SA/Def-s-TiO_2	13.5	10	300	2021	30
Cu_3P@NiCoP	8.9	5	5	2021	39
3D porous carbon nitride	17	16	300	2021	31
Monodispersed ZnS/NiO	162	8	300	2021	34
Pt-BP/CdS	24.2	20	300	2021	28
CdS–ZnO core–shell	268.5	18	300	2021	35

Supplementary table 2 ICP-OES analysis of the AuNP@CoTPyP structures prepared at different concentrations of CoTPyP.

C(CoTPyP)	2000 nM	200 nM	20 nM	2 nM
**Co:Au**	9.2 : 1000	9.1 : 1000	6.4 : 1000	1 : 1600

Supplementary table 3 Frontier orbital energies for CoTPyP and AuNP@CoTPyP.

	CoTPyP	AuNP@CoTPyP
**HOMO**	-6.00	-6.19
**LUMO**	-2.76	-2.97
